# Novel Observations Concerning Differentiation of Bloodstream-Form Trypanosomes to the Form That Is Adapted for Growth in Tsetse Flies

**DOI:** 10.1128/mSphere.00533-18

**Published:** 2018-10-31

**Authors:** Christine Clayton

**Affiliations:** a Centre for Molecular Biology of Heidelberg University (ZMBH), DKFZ-ZMBH Alliance, Heidelberg, Germany; University of Georgia

**Keywords:** RNA splicing, *Trypanosoma*, mRNA degradation, translation

## Abstract

Salivarian trypanosomes grow in mammals, where they depend on glucose, and as procyclic forms in tsetse flies, where they metabolize proline. Differentiation of bloodstream forms to nongrowing stumpy forms, and to procyclic forms, has been studied extensively, but reconciling the results is tricky because investigators have used parasites with various differentiation competences and different media for procyclic-form culture. Standard protocols include lowering the temperature to 27°C, adding a tricarboxylic acid, and transferring the parasites to high-proline medium, often including glucose. A 20°C cold shock enhanced efficiency. Y. Qiu, J. E. Milanes, J. A. Jones, R. E. Noorai, et al. (mSphere 3:e00366-18, 2018, https://doi.org/10.1128/mSphere.00366-18) studied this systematically, and their results call long-established protocols into question. Importantly, highly efficient differentiation was observed after cold shock and transfer to no-glucose medium without tricarboxylic acid; in contrast, glucose made differentiation tricarboxylic acid dependent and inhibited procyclic growth. New transcriptome data for stumpy and procyclic forms will enable informative comparisons with biochemical observations and with other RNA and protein data sets.

## Abstract (precis)

Salivarian trypanosomes grow in mammals, where they depend on glucose, and as procyclic forms in tsetse flies, where they metabolize proline. Differentiation of bloodstream forms to nongrowing stumpy forms, and to procyclic forms, has been studied extensively, but reconciling the results is tricky because investigators have used parasites with various differentiation competences and different media for procyclic-form culture.

## COMMENTARY

Salivarian trypanosomes are elongated, flagellated unicellular eukaryotes which multiply extracellularly in the blood and tissue fluids of mammals. They are transmitted by tsetse flies, where they occupy the digestive tract. Two Trypanosoma brucei subspecies cause sleeping sickness in humans; other Salivaria, including *Trypanosoma brucei brucei*, infect ruminants. The human disease is currently well controlled, but cattle trypanosomosis causes immense economic losses. The parasites escape adaptive immunity by antigenic variation, and the few available drugs have numerous deficiencies, but efforts to develop new therapies are beginning to bear fruit.

“Bloodstream-form” T. brucei is cultured at 37°C in medium containing 5 mM glucose, mimicking conditions inside mammals. Bloodstream forms have a reduced mitochondrion and rely on glycolysis for ATP generation. Multiplying bloodstream forms have long slender morphology, but at high density, quorum sensing prompts them to become growth-arrested “short stumpy” forms (1). These express additional mitochondrial proteins and other markers. After infected blood enters the fly, glucose is rapidly depleted and the parasites transform to procyclic forms. Procyclic forms express surface procyclins and have an elaborate mitochondrion with active oxidative phosphorylation, which enables them to use the substrates available—predominantly proline—for ATP formation. Continuous laboratory passage or culture of bloodstream forms selects strongly against stumpy formation, resulting in “monomorphic” lines. These usually retain the ability to express some typical procyclic-form markers when subjected to appropriate stimuli, but growth as procyclic forms is either absent or extremely inefficient.

The slender-to-stumpy differentiation process has been intensively studied and is now partially understood (2). The paper from Qiu et al. (3), in contrast, focuses on the next step, differentiation to procyclic forms. Relevant results in the extensive prior literature are quite difficult to disentangle, because investigators have used not only trypanosomes with wildly different passage histories and differentiation capabilities but also various media for procyclic-form cultivation. In particular, in addition to 5 mM proline, glucose was present at either 500 µM (“low glucose”) or 5 mM (“high glucose”). Temperature reduction to 27°C is clearly a major differentiation trigger, along with addition of 6 mM citrate or *cis*-aconitate (4, 5). Preincubation at 20°C (cold shock) increased the expression of a putative tricarboxylic acid receptor (6), the sensitivity to tricarboxylic acids, and the differentiation efficiency (7). However, glucose starvation of monomorphic parasites also triggered some of the changes required for the switch to procyclic forms (8, 9).

Qiu et al. (3) now describe an extensive series of experiments to dissect the effects of glucose, cold shock, and citrate on both differentiation of stumpy-form parasites and their subsequent growth as procyclic forms. They purified long slender or stumpy forms from infected mice, cultured them for 1 day in bloodstream-form medium at 37°C (allowing recovery from purification), and then transferred them to alternative procyclic-form media at 27°C. Importantly, stumpy forms (but not long slender forms) differentiated quite well if they were simply transferred to procyclic media containing 5 µM glucose (here called “no glucose”). In contrast, as previously seen, when stumpy forms were transferred to high-glucose procyclic medium, differentiation could be obtained only after addition of 6 mM citrate or, after a cold shock, of low citrate (16 µM, physiological for tsetse flies). A very novel observation was that the best differentiation efficiency and procyclic growth were observed when cells were transferred to no-glucose procyclic medium and cold shocked. The collected data also suggest that the effect of cold shock may actually not be due to an increase in carboxylic acid sensitivity, as previously thought; instead, it may allow a larger proportion of the cells to retain viability during the early stages of adaptation to procyclic conditions. This warrants further investigation.

The authors show at least one reason why stumpy forms are required for tsetse fly transmission: unlike long slender forms, they have sufficient oxidative phosphorylation to survive for 2 days with proline instead of glucose—more than sufficient time for procyclic differentiation. As expected (10), all procyclic form cultures were absolutely dependent on proline. Experiments using glucose analogues and inhibitors suggested that glucose-related signaling pathways, as well as metabolic effects, might influence procyclic growth, but without metabolite measurements (including effects on gluconeogenesis [11]), the results are difficult to interpret.

Very usefully, the culture data are supplemented by transcriptomes, enabling comparisons of stumpy forms with long slender bloodstream forms and no-glucose procyclic forms. To my knowledge, these are currently the only RNASeq transcriptomes available for rodent-derived stumpy forms. Interestingly, although the downregulated genes are quite similar to those previously seen in *in vitro*-generated stumpy forms (12), there is little correlation for the upregulated mRNAs. Here, too, more investigation is required. Stumpy-form preincubation with proline had little effect on the transcriptomes, which is not unexpected since their transcription is suppressed, but perhaps translation or posttranslational modifications are affected?

Procyclic forms are known to adapt to low- or high-glucose media: for example, glucose-adapted parasites use it in preference to (but not to the exclusion of) proline (10, 13) while low-glucose-adapted procyclic forms have higher rates of proline uptake (14) and grow to higher densities (14). Qiu et al. (3) indeed observed that the growth rate of procyclic forms that had been grown in high glucose increased when glucose was completely withdrawn. Usefully, they also compared transcriptomes of procyclic forms from these two conditions. The only other similar comparison available is for ribosome footprints from procyclic forms grown in either high or low glucose (15), but there, many other components of the media were also different. Nevertheless, some observations were common to both studies. Although the authors compared their transcriptomes with several others, comparisons with additional data sets, and with metabolic observations, will be even more informative.

Procyclic media with unphysiologically high glucose levels have been used routinely in many laboratories for several decades. The results of the work by Qiu et al. (3) clearly show that low- or no-glucose media are preferable and suggest that some conclusions regarding trypanosome differentiation need to be reassessed.
